# Effects of larval density on dispersal and fecundity of western corn rootworm, *Diabrotica virgifera virgifera* LeConte (Coleoptera: Chrysomelidae)

**DOI:** 10.1371/journal.pone.0212696

**Published:** 2019-03-01

**Authors:** Eric Yu Yu, Aaron J. Gassmann, Thomas W. Sappington

**Affiliations:** 1 Department of Entomology, Iowa State University, Ames, Iowa, United States of America; 2 Corn Insects & Crop Genetics Research Unit, USDA-Agricultural Research Service, Ames, Iowa, United States of America; Zhejiang University, CHINA

## Abstract

The western corn rootworm, *Diabrotica virgifera virgifera* (LeConte) (Coleoptera: Chrysomelidae), is an economically important pest of corn in the northern United States. Some populations have developed resistance to management strategies including transgenic corn that produces insecticidal toxins derived from the bacterium *Bacillus thuringiensis* (Bt). Knowledge of insect dispersal is of critical importance for models of resistance evolution. Larval density affects survival in the field, and stress from crowding often affects facultative long-distance dispersal of adult insects. In this study, we used laboratory flight mills to characterize western corn rootworm flight performance as a function of larval rearing density. Larvae were reared under three densities and the resulting adult females were either allowed to fly voluntarily for 22 h or forced to fly specified durations. For both experiments we also measured lifetime fecundity following flight. The three rearing densities placed differential levels of stress on individuals, as evidenced by decreased survival to adulthood and decreased size of adults at greater rearing density. When larvae were reared under crowded conditions the resulting females were more likely to engage in flight activity, including long uninterrupted flights lasting >10 min, than those reared under low density conditions. Flight and egg production are both energy intensive processes. However, we found no evidence in either voluntary or forced flight experiments of a tradeoff between flight activity and female fecundity. The results suggest that females emerging from high density populations in cornfields are more likely to disperse and disperse farther than those emerging from low density populations. These results are important because they imply that variation in population density in the landscape will affect dispersal, which may in turn require computer models of resistance evolution to incorporate multiple dispersal rates arising from varying larval densities among fields.

## Introduction

The western corn rootworm, *Diabrotica virgifera virgifera* LeConte (Coleoptera: Chrysomelidae), is a serious pest of corn (*Zea mays* L.) in the United States. This pest is univoltine, with a life cycle tightly linked to corn, its primary larval host [1]. In the U.S. Corn Belt, adults begin to emerge in late June with peak emergence during July [2, 3]. Oviposition takes place almost exclusively in cornfields from July through mid-September. The eggs undergo obligate diapause through the winter, and typically hatch during late spring [4]. Both larval and adult stages are capable of damaging corn, but larvae cause more yield loss. Adult rootworm feed on corn foliage, pollen, silks, and developing kernels [5]. Larvae feed on corn roots, which can reduce absorption of water and nutrients [6]. Extensive root injury also makes plants more susceptible to lodging, leading to yield loss by complicating harvest [7]. Fifteen percent yield loss is associated with every node of roots pruned by larvae [8].

Transgenic corn hybrids that express *Bacillus thuringiensis* (Bt) toxins have been widely planted (77% of U.S. corn in 2017) to manage targeted insect pests [9]. Bt corn, targeting western corn rootworm, was first commercialized in 2003 [10]. Currently, four Bt toxins produced in corn, either singly or as a pyramid, target western corn rootworm larvae: Cry3Bb1, Cry34/35Ab1, mCry3A, and eCry3.1Ab [11, 12].

The western corn rootworm is highly adaptable, and populations have evolved resistance to several management strategies, including transgenic Bt corn [13]. The rapid adoption of Bt corn led to field-evolved Bt resistance in western corn rootworm, first identified in Iowa cornfields in 2009 [14]. By 2016, field-evolved resistance to all four commercially available Bt toxins had been demonstrated [15–17]. Dispersal by individuals is the fundamental process by which resistance genes move across a landscape [18]. Resistance alleles can spread through an established population or can be carried by individuals colonizing new areas. Postmating dispersal of western corn rootworm by flight will affect the spatial spread of resistance from local hotspots to the surrounding landscape [19].

Extensive efforts have been made to characterize the flight behavior of western corn rootworm. Within the first few hours of emergence, females move only short distances before they are intercepted by mate-seeking males [20, 21]. After mating, females remain in their natal cornfield to feed for several days before dispersing to neighboring fields [22]. Females are more abundant than males among western corn rootworm flying between fields [23]. Although males fly extensively in cornfields to locate mates, they are less likely to initiate long-distance flight than females [24]. Flights between fields typically occur during morning and evening hours [25, 26]. Long-distance dispersal is evidenced by mass depositions of western corn rootworm in the Great Lakes [27], ascent of females above the atmospheric boundary layer [22], and rapid colonization of first-year cornfields [28].

In this study, we used flight mills to examine flight behavior of western corn rootworm females. Flight mills, which constrain tethered insects to fly in a horizontal circle, are often used to study insect flight behavior and capacity. Although flight mills provide a simple way to evaluate the flight potential of insects, there are complications in translating tethered flight results to natural flight in the field. An insect on a flight mill is suspended and provided vertical support of its weight, negating the need to expend energy to provide lift during flight [29]. On the other hand, a tethered insect must provide additional thrust to overcome pivotal friction and aerodynamic drag from the flight mill arm [30]. Furthermore, natural flight can occur above the boundary layer, where flight speed is strongly enhanced by wind [31]. Despite these limitations, flight mills are important tools for studying how various biotic and abiotic factors may influence an insect’s propensity to disperse [32–35].

Larval density affects survival of western corn rootworm in the field [36–38], and, in many insect species, stress from larval crowding facultatively increases long-distance dispersal of adults [39]. We predicted that adults emerging from high larval densities would have greater flight propensity and performance than those reared at low density. Furthermore, flight is an energetically costly behavior that may compete with adult reproduction [40, 41]. We predicted that female western corn rootworm adults that engaged in longer flights would produce fewer eggs than those making shorter flights.

## Materials and methods

### Experimental design

We conducted two experiments with western corn rootworm to measure the effects of larval density on adult flight activity and whether a tradeoff exists between flight and fecundity. These experiments included 1) voluntary flight and 2) forced flight. For the voluntary flight experiments, we reared western corn rootworm at three different larval densities. Females were allowed to mate upon adult emergence. Flight mills were then used to explore density-dependent effects of larval crowding on voluntary flight behavior of 6 day old female adults. Flight-tested females were allowed to mate and oviposit until death to assess lifetime fecundity. Additionally, 6 day old females reared at a single larval density were forced to fly specified lengths of time to test the effect of flight activity on fecundity, without the potentially confounding effect of density on flight propensity. The specific goals of this study were to measure the effects of larval density on flight performance of females, and to examine the effects of flight on female lifetime fecundity.

### Collecting eggs from western corn rootworm adults

To obtain western corn rootworm eggs for these experiments, adult beetles were collected with an aspirator from cornfields at two Iowa State University research farms located in Ames, Iowa and Nashua, Iowa. During the summers of 2015 and 2016, approximately 500 western corn rootworm beetles were collected from each location. Beetles were placed in mesh cages (18×18×18 cm, MegaView Science Co. Ltd., Taichung, Taiwan) and held in separate cages by population in an incubator (Percival Scientific, Perry, IA) at 25° C and 60% RH.

Caged adults were provided chopped corn ear, corn leaf tissue, and 1.5% agar solid (Dehydrated agar powder, Fisher Scientific, Pittsburgh, PA). Moistened sieved field soil (<180 μm) was placed in a Petri dish (100 mm×15 mm, Fisher Scientific, Pittsburgh, PA) as an oviposition substrate. Eggs were collected and incubated for one month at 25° C and 60% RH. After incubation, eggs were washed in a 250-μm sieve (Fisher Scientific, Hampton, NH) to remove soil. Cohorts of approximately 1,500 eggs were placed in cold storage (6° C) to diapause for at least 6 months. After this time a single cohort of eggs was removed weekly, alternating populations, to provide a steady supply of neonates for experiments. Ten-thousand and 50,000 eggs were collected from each field population during the summers of 2015 and 2016, respectively, to supply insects for the first and second year of experiments. All life stages were held at 25° C, 60% RH and 14:10 (L:D) photoperiod, except eggs in cold storage.

### Rearing western corn rootworm larvae

Larvae were reared following methods modified from Ingber and Gassmann [42]. Neonates typically hatched 15 days after removal from cold storage. A soft bristle brush was used to transfer 12 neonates onto roots of three germinated corn kernels in a 44-ml container (Dart, Mason, MI). 40 ml of moistened sieved soil (<600 μm) was placed over the infested germinated kernels, and mesh fabric (Chiffon; Hobby Lobby Stores, Inc. Oklahoma City, OK) covered the container to prevent larval escape. Each infested container was held in a growth chamber for 7 days, then transferred to a 473-ml container (Placon, Madison, WI). The latter contained a specified number (depending on density treatment) of corn kernels planted in a 120-g mixture of 50% sieved field soil (<600 μm) and 50% potting soil (Sun Gro Horticulture, Agawam, MA) moistened with 20 ml deionized water. To achieve the high density treatment, contents of two 44-ml containers were transferred to a single 473-ml container, while low and moderate density treatments received one 44-ml container. Larvae were reared under three density treatments, imposed as larvae per seedling, beginning 7 days after hatch. Low density: 12 larvae per 36 seedlings (0.33 larvae per seedling); moderate density: 12 larvae per 10 seedlings (1.2 larvae per seedling); and high density: 24 (i.e., from two 44-ml containers) larvae per 5 seedlings (4.8 larvae per seedling).

Adults emerging from the density treatments were collected daily using an aspirator (BioQuip, Rancho Dominguez, CA) attached to a vacuum (Gast Manufacturing, Inc., Benton Harbor, MI) and segregated by date and sex. To determine sex, each beetle was anesthetized with CO_2_ and examined under a dissecting microscope for morphology of the prothoracic basitarsi [43]. The number of emerging adults per container was recorded to assess proportion survival to adulthood. Adults were maintained in mesh cages as described earlier. Males 7–14 days old were placed in each cage of newly emerged females at a ratio of 3:2 to promote mating. Females are sexually mature upon adult emergence [44], whereas males require 5 to 7 days of post-emergence development to reach sexual maturity [45]. Females were flight-tested 6 days after emergence, the age of peak sustained flight behavior [34].

### Measuring flight of western corn rootworm

Flight tests were conducted using 16 flight mills housed in an environmental chamber with programmable lighting, temperature, and humidity control (Percival Scientific, Perry, IA; 25° C, 60% RH). Air flow in the chamber was directed through baffled wall panels to ensure even temperature distribution without creating air currents. The chamber was equipped with eight 35W LED modules consisting of four high output cool white LEDs and three high output red LEDs. The photoperiod was 14:10 (L:D), matching natural day length in early August in central Iowa. The 14 h of daylight included 30 min of simulated dawn where light intensity ramped up gradually from fully off to fully on, and the 10 h of dark included 30 min of simulated dusk. Red LEDs were left on continuously, and white LEDs were on during the 14-h light cycle. The light-dark cycle was offset from local time by +3 h to accommodate working hours. Sunrise during early August in central Iowa is around 07:00 h, and sunset is around 21:00 h. Under lab conditions, sunrise was set at 10:00 h, and sunset at 00:00 h.

Flight mills were custom built and modified slightly from the design by Jones et al. [46] (S1 Fig). Each flight mill has two opposing magnets, separated by a hypodermic thin wall tube, which generates a levitation effect to minimize friction at the pivot of the flight arm. A 31.8-cm long hypodermic tube served as the flight arm which passed through the top center of the Teflon bearing. The tethered insect was attached to one end of the flight arm. As the insect flew in circles (1 m circumference), a small magnet suspended below the Teflon bearing was detected by a digital Hall-effect sensor. Wires from each flight mill ran to a main connector board and plugged into a PC port. The data were collected and analyzed with a custom flight mill software program modified from Jones et al. [46] using LabVIEW (National Instruments, Austin, TX). The raw data were exported to a spreadsheet as a .csv text file and output included the total number of flights, and the time of day, distance, and duration of each individual flight.

Methods to tether adults were modified from Stebbing et al. [47]. Adults were anesthetized with CO_2_ for <15 sec which allowed up to 1 min for tethering without insect movement. A short length of galvanized steel wire (28 gauge, The Hillman Group, Cincinnati, OH) was bent 90° and one end attached to the pronotum with a small bead of warmed dental wax (DenTek, Maryville, TN). The free end of the wire was inserted into the opening of the hypodermic tube serving as the flight mill arm, thus suspending the tethered beetle. Each insect was provided a small piece of tissue paper (Kimwipe, Kimberly-Clark, Irving, TX) for tarsal contact, which helped reduce premature flight before dawn. After completion of a flight mill test, the wax bead connecting the tether to the pronotum was removed, and females were maintained until death to measure lifetime fecundity. The wax separated easily from the pronotum with no apparent damage to the cuticle.

### Voluntary flight experiments

Voluntary flight experiments were conducted during 2016 and 2017. Six day old females were tethered in the flight mill chamber under red lighting approximately 1–2 h before simulated dawn to allow a full 14 h of daylight for flight activity. Adults were removed from flight mills 22 h later, before simulated dawn. Voluntary flight behavior during the 22 h test period was characterized for total flight activity and for the longest uninterrupted flight by each individual. In migratory insects, the longest uninterrupted flight sometimes represents the straight-line flight behavior characteristic of migratory flight [48]. Females that made at least one continuous flight of ≥1 min were placed in an oviposition container with a single male from the same density treatment and location. A total of 307 females, across all densities and locations, were flight tested. Of those, 49% did not meet the minimum criterion for flight (see ‘Data Analysis’). For those meeting the minimum flight duration, adult sample sizes for larval density treatments were as follows for Ames: Low = 23, Moderate = 28, High = 18; and for Nashua: Low = 31, Moderate = 32, High = 26.

### Forced flight experiments

Forced flight experiments were conducted during 2017 throughout the 14 h daylight period. Six day old females from the Ames population reared under moderate larval density, as previously described, were tested on flight mills. Adults were assigned one of four flight duration treatments: 10, 30, 60, and 120 min duration of forced flight. The flight mill software provided precise data on the flight activity of each adult tested. Flight was stimulated with a stream of air from an airline tube attached to an aquarium air pump (20–60 gal, Aqua Culture, Bentonville, AR). A non-flying female received a 5-second stream of air, followed by a 20-second break if flight was not initiated, after which a stream of air was reapplied. Females that ceased flying were stimulated to reinitiate flight throughout the test period. Each flight-tested beetle was accompanied by a paired control beetle, which was tethered to the bottom of the same flight mill but positioned to make tarsal contact on the shelving surface to prevent flight. The control beetle received a stream of air from a second tube simultaneously with the flight-tested individual. Females that did not engage in flight during the testing period were discarded, along with their paired control. We tested 30 females per treatment (total N = 120 flight and 120 control insects each) but sample sizes were reduced based on a minimum-flight duration threshold (see ‘Data analysis’). Thus, final paired samples sizes were 19 for 10 min (i.e., 19 flown + 19 control), 14 for 30 min, 10 for 60 min, and 12 for 120 min.

### Fecundity

A 473-ml oviposition container was designed to house male-female pairs, chopped corn ear for food, and oviposition substrate. The container was ventilated through the lid by a hole covered with fiberglass insect screen (20 x 20 μm mesh, Phifer, Tuscaloosa, AL). The bottom of the container was covered in moistened sieved field soil (<180 μm) textured with grooves to stimulate oviposition. Additional 3-dimensional structure was added by placing pieces of soil onto the grooved surface. This was achieved by mixing sieved field soil (<180 μm) with deionized water and allowing the mixture to dry. The resulting block of soil was broken apart and mounded on top of the textured layer. Chopped corn ear was held in a Petri dish (55 mm diameter) suspended from the lid with twist ties. Each female was housed in the oviposition container with a single male until death. Oviposition containers were checked daily for insect mortality. Every 2 days diet was changed and 10 ml of deionized water was sprayed into the container through the screened hole. Dead males were replaced, and dead females were preserved in 85% ethanol.

Upon death of the female, soil in the oviposition container was washed from eggs through a 250-μm sieve. All eggs produced by a female were placed in a 1.5 ml micro-centrifuge tube containing 85% ethanol, and counted later under a dissecting microscope (Leica Mz6, Leica Microsystems, Wetzlar, Germany) to quantify lifetime fecundity.

### Morphology

Morphological measurements were obtained from digital photos of females taken with a dissecting microscope fitted with a digital camera (Moticam 2500, Motic Images, Inc., Richmond, British Columbia, Canada) and accompanying image analysis software (Motic Images Plus 2.0; Motic Images, Inc.). Elytron length and head capsule width were measured to quantify size. Elytron length was measured at the greatest distance between the apex and base. The head capsule was measured as the distance between the distal most points of each eye.

### Data analysis

For voluntary and forced flight experiments, individuals were excluded from analyses if they did not meet minimum criteria for flight performance, fecundity, and longevity. This was to exclude insects that may have been damaged during handling or were in poor health. To be included in voluntary flight analyses, females must have made at least one continuous flight lasting ≥1 min. The longest single flight was examined only for females that made a continuous flight lasting at least 10 min. This threshold excluded approximately 70% of tested beetles and was used to prevent the large number of short flights from swamping any signal from longer flights that could represent distinctive long-distance dispersal behavior. Additionally, to be included in analyses, beetles must have lived for at least 3 days following flight to reduce the number of beetles whose health was compromised by experimental conditions. Finally, a female must have oviposited at least one egg during its lifetime to eliminate unmated beetles or mated beetles incapable of reproduction.

All statistical analyses were conducted using SAS Enterprise Guide 7.1 (SAS Institute, Cary, NC). For voluntary flight experiments, data on proportion survival to adulthood, size, and flight parameters were compared among larval density, location, and interaction of density and location with a model I analysis of variance (ANOVA) (PROC GLM), with larval density treated as a quantitative variable. To ensure normality of residuals, flight data were transformed by the log_10_ function.

Linear regression analysis (PROG REG) was used to test the effect of flight on lifetime fecundity for voluntary and forced flight experiments. In the forced flight experiments, not all females flew the entire intended duration, and there was extensive overlap in the distribution of actual flight durations between treatment groups. To address this, females that did not fly at least 75% of the intended treatment duration were excluded, which created a separation among the treatments.

## Results

**Fig 1:**
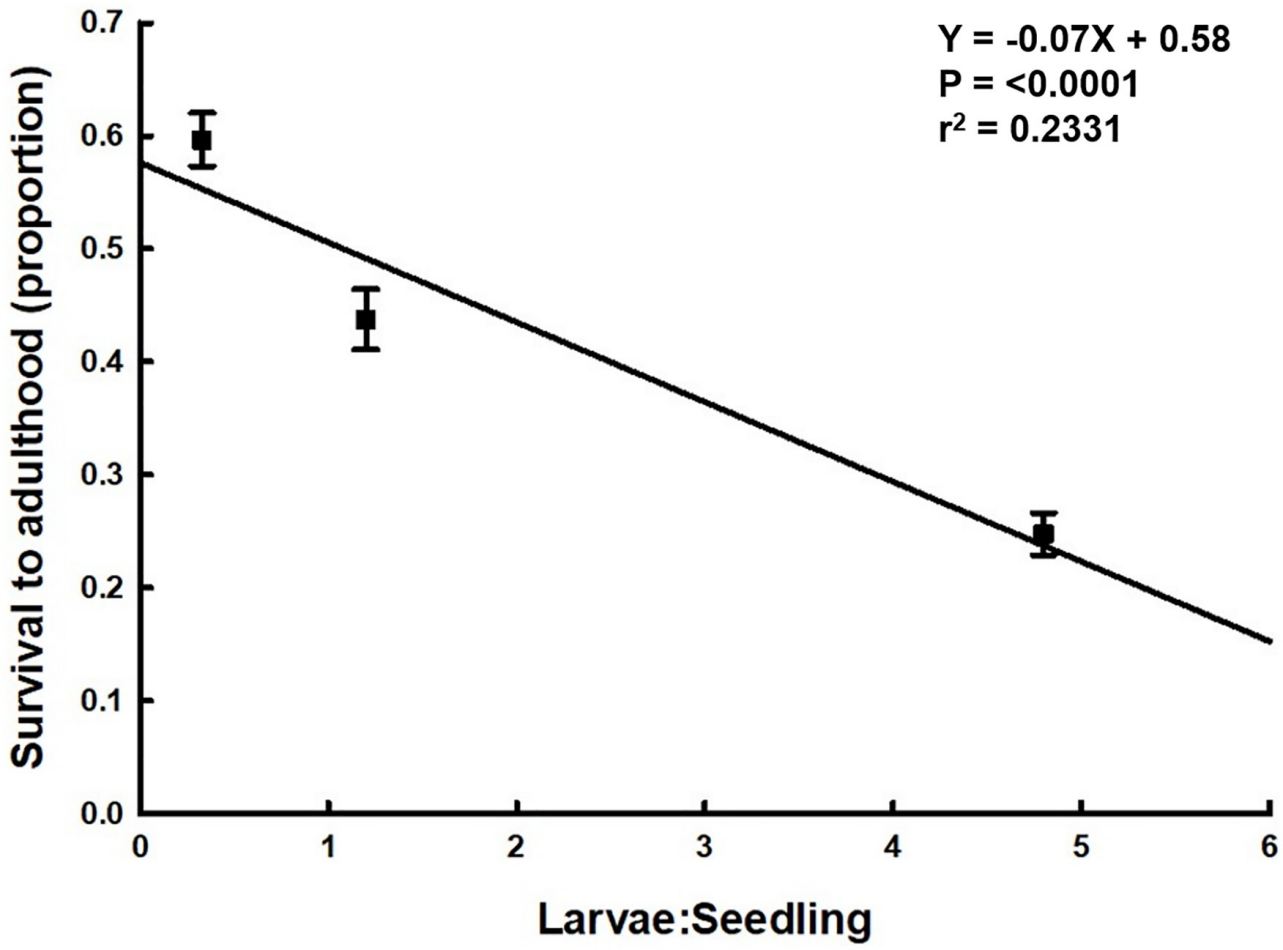
Survival to adulthood of western corn rootworm males and females reared at different larval densities. Points represent sample means, and error bars indicate SEM. Linear regression analysis was conducted with raw data. Linear regression of proportion adult emergence (Y) on larval density (X): df = 1, 370, Y = -0.07X + 0.58, t = -10.61, P <0.0001, r^2^ = 0.2331.

**Fig 2:**
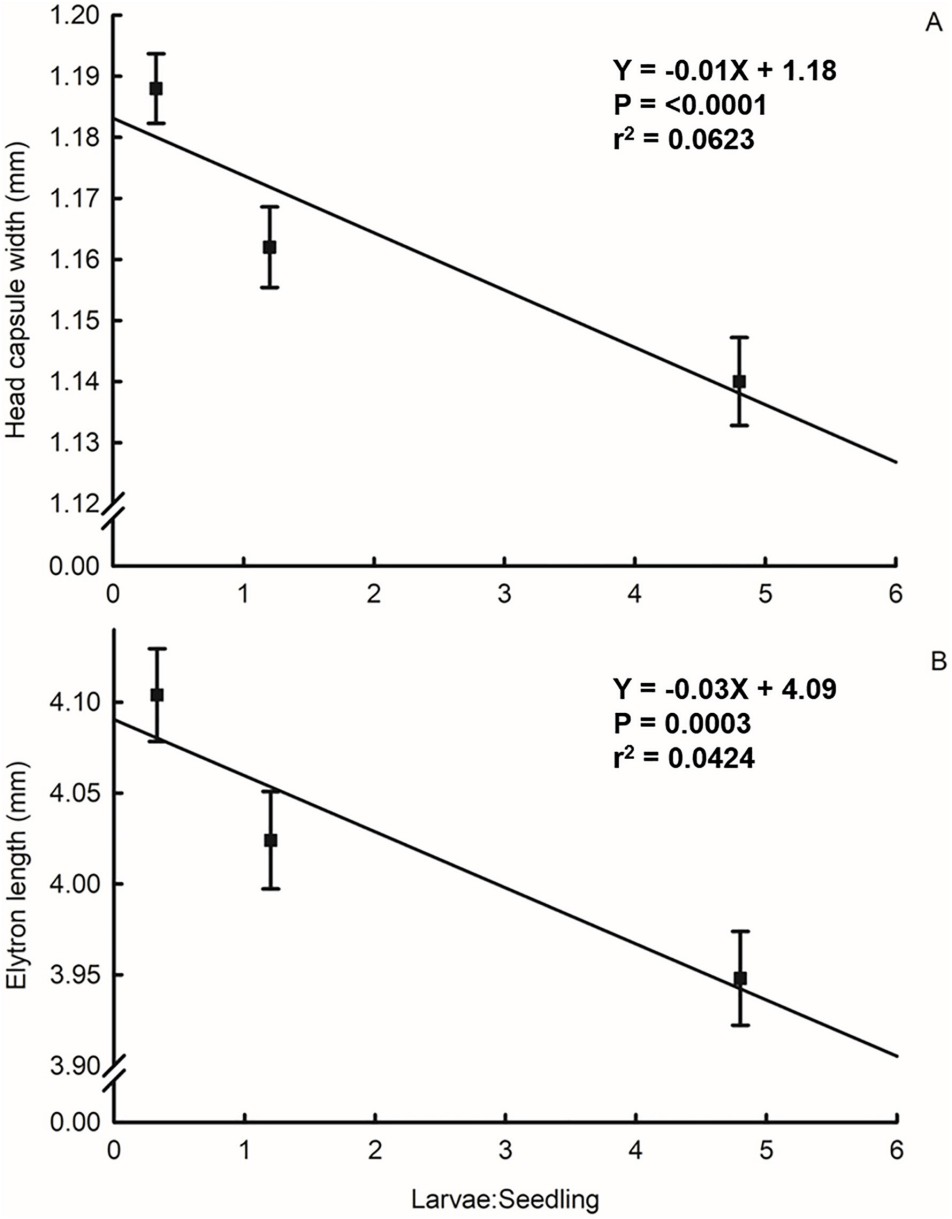
Size of western corn rootworm adults reared at different larval densities measured by (A) head capsule width, and (B) elytron length. Points represent sample means, and error bars indicate SEM. Linear regression analyses were conducted with raw data. Linear regression of morphological measurements (Y) on larval density (X): (A) df = 1, 305, Y = -0.01X + 1.18, t = -4.50, P <0.0001, r^2^ = 0.0623; (B) df = 1, 305, Y = -0.03X + 4.09, t = -3.68, P = 0.0003, r^2^ = 0.0424.

Because there were no significant effects of location or interaction between density and location on any of the variables examined (Tables 1 and 2), data from both locations were combined in graphical presentations. A density-induced stress gradient was successfully imposed on larvae, as evidenced by the effects of larval density on adult emergence and size. Proportion survival to adulthood significantly decreased with increasing rearing density (Fig 1; Table 1), while head capsule width and elytron length significantly decreased (Fig 2; Table 1).

**Table 1:** Analyses of variance for life-history characteristics from the voluntary flight experiment.

Analysis	Effect	df	F	P
Survival to adulthood^a^	Density^b^	1, 368	110.42	<0.0001
	Location^c^	1, 368	1.45	0.2288
	Density × Location	1, 368	2.62	0.1064
Head capsule width	Density	1, 303	20.22	<0.0001
	Location	1, 303	0.94	0.3321
	Density × Location	1, 303	1.08	0.3003
Elytron length	Density	1, 303	13.38	0.0003
	Location	1, 303	0.08	0.7726
	Density × Location	1, 303	1.79	0.1821
Longevity (days)	Density	1, 303	0.10	0.7495
	Location	1, 303	0.52	0.4698
	Density × Location	1, 303	1.25	0.2646
Egg number per female	Density	1, 303	1.74	0.1878
	Location	1, 303	3.56	0.0601
	Density × Location	1, 303	0.36	0.5498

^a^Proportion survival to adulthood

^b^Larval rearing density

^c^Site of collection (i.e., Ames or Nashua)

**Table 2:** Analyses of variance for flight data from the voluntary flight experiment.

Analysis	Effect	df	F	P
Total flight distance^a^	Density^c^	1, 154	8.30	0.0045
	Location^d^	1, 154	0.61	0.4369
	Density × Location	1, 154	0.64	0.4252
Longest single flight distance^a^^b^	Density	1, 41	2.74	0.1054
	Location	1, 41	4.23	0.0461
	Density × Location	1, 41	3.05	0.0882
Total flight duration^a^	Density	1, 154	9.85	0.0020
	Location	1, 154	0.90	0.3438
	Density × Location	1, 154	1.02	0.3136
Longest single flight duration^a^^b^	Density	1, 41	4.15	0.0481
	Location	1, 41	4.21	0.0467
	Density × Location	1, 41	1.95	0.1699
Total flight speed	Density	1, 154	1.75	0.1879
	Location	1, 154	0.05	0.8298
	Density × Location	1, 154	0.42	0.5197
Longest single flight speed^b^	Density	1, 41	0.39	0.5335
	Location	1, 41	0.79	0.3802
	Density × Location	1, 41	3.19	0.0813

^a^Raw data were log-transformed

^b^Longest single flight parameter excluded individuals that did not engage in a single uninterrupted flight of at least 10 min

^c^Larval rearing density

^d^Site of collection (i.e., Ames or Nashua)

**Fig 3:**
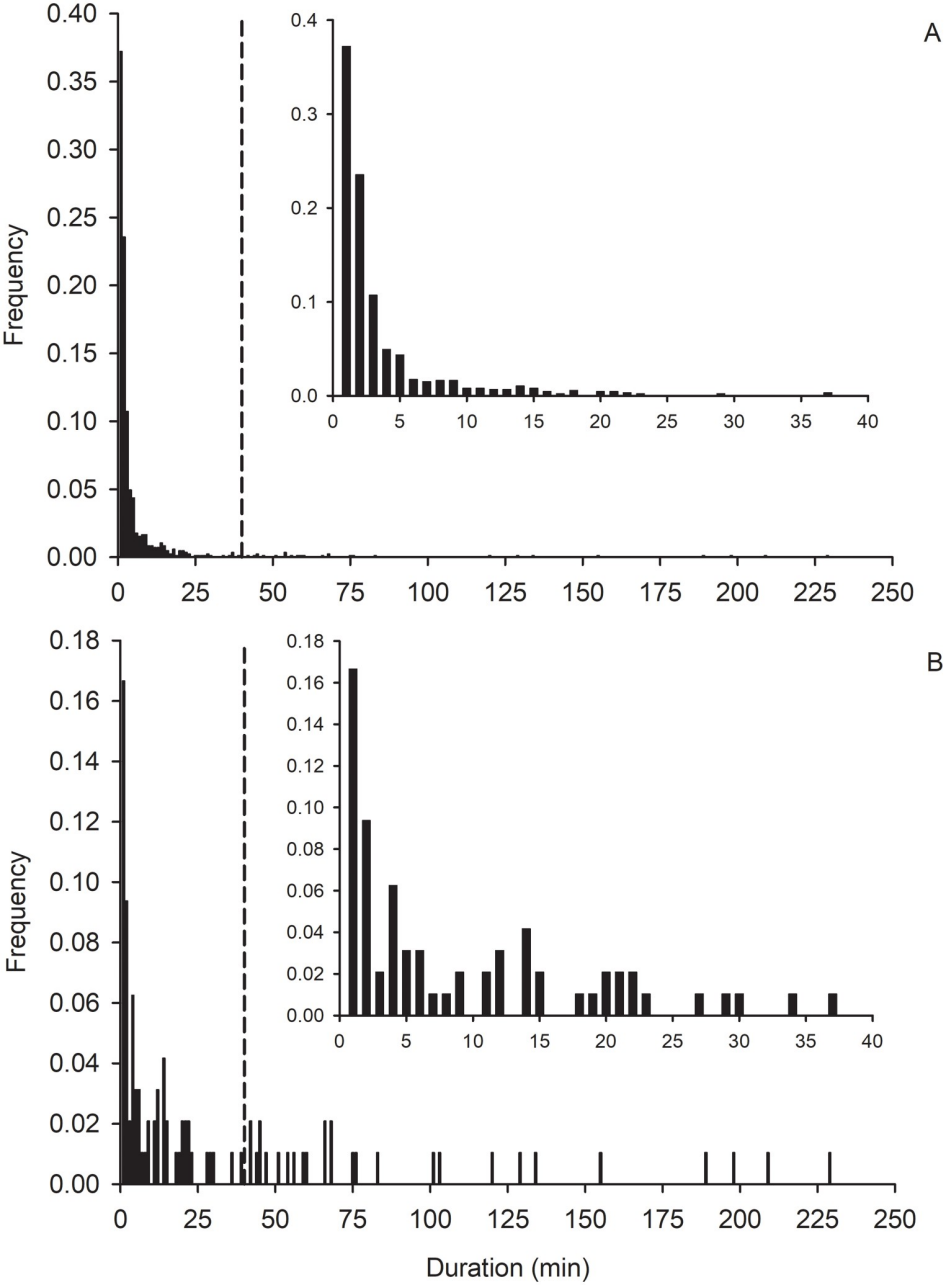
Frequency distribution of durations of (A) all flights, and (B) longest single flights by female western corn rootworm in the voluntary flight experiment. Dashed line marks 40 min. Inset figures represent frequency of durations from 1 min to 40 min of flight. Flights lasting <1 min were excluded.

Combining data across densities, we examined the distribution of all individual flight durations by each beetle in the voluntary flight experiment (Fig 3). Of the 307 females tested, 158 engaged in flight. These beetles flew a total of 2,397 flights, 1,548 of which lasted <1 min and were excluded from analysis. The distributions were skewed with 70% of flights lasting ≤3 min. Distributions, for total flight activity and longest single flights, declined rapidly as flight duration increased. There was no clear break in the frequency distribution of flights between 20 or 30 min as described from previous tethered flight experiments with western corn rootworm [34, 49]. However, the frequencies of durations of all flights and longest single flights had reached a low level by 10 min (Fig 3). Therefore, trivial flights were defined as individual flights lasting <10 min and sustained flights were defined as individual flights lasting ≥10 min. All analyses that included longest single flight excluded females that did not make a single uninterrupted flight of at least 10 min.

**Fig 4:**
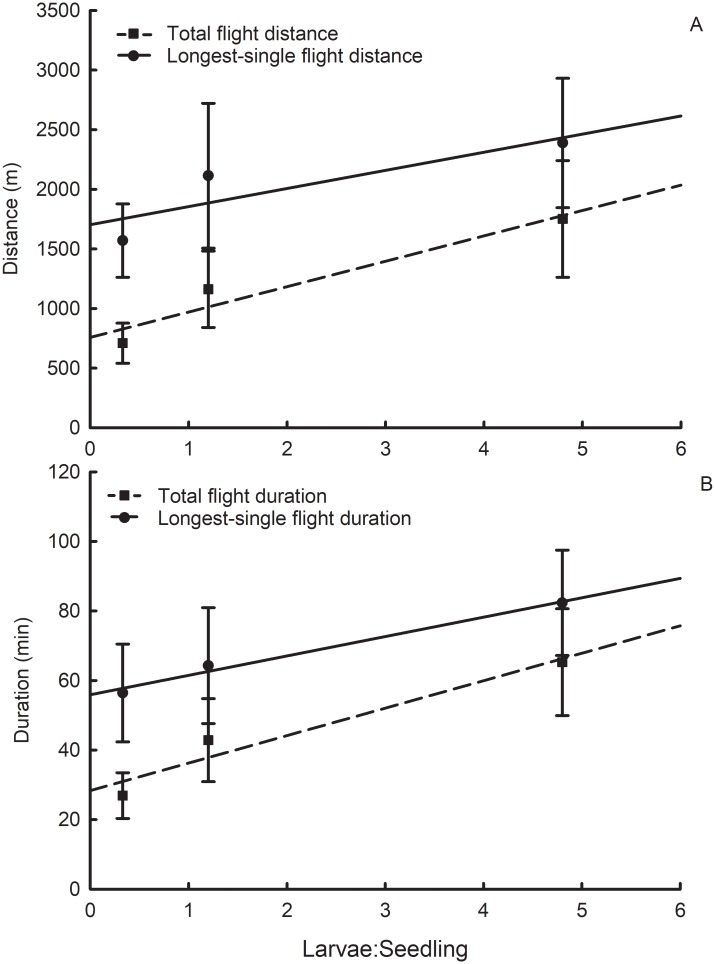
Influence of western corn rootworm larval rearing densities on (A) flight distance, and (B) flight duration, in the voluntary flight experiment. Points represent sample means, and error bars indicate SEM. Linear regression analyses were conducted with raw data. Linear regression of flight parameters (Y) on larval density (X): (Total flight distance) df = 1, 156, Y = 213.29X + 765.55, t = 2.05, P = 0.0421, r^2^ = 0.0262; (Longest single flight distance) df = 1, 43, Y = 109.60X + 1738.07, t = 0.72, P = 0.4784, r^2^ = 0.0118; (Total flight duration) df = 1, 156, Y = 7.84X + 29.10, t = 2.18, P = 0.0304, r^2^ = 0.0297; (Longest single flight duration) df = 1, 43, Y = 4.47X + 56.91, t = 0.99, P = 0.3290, r^2^ = 0.0222.

**Fig 5:**
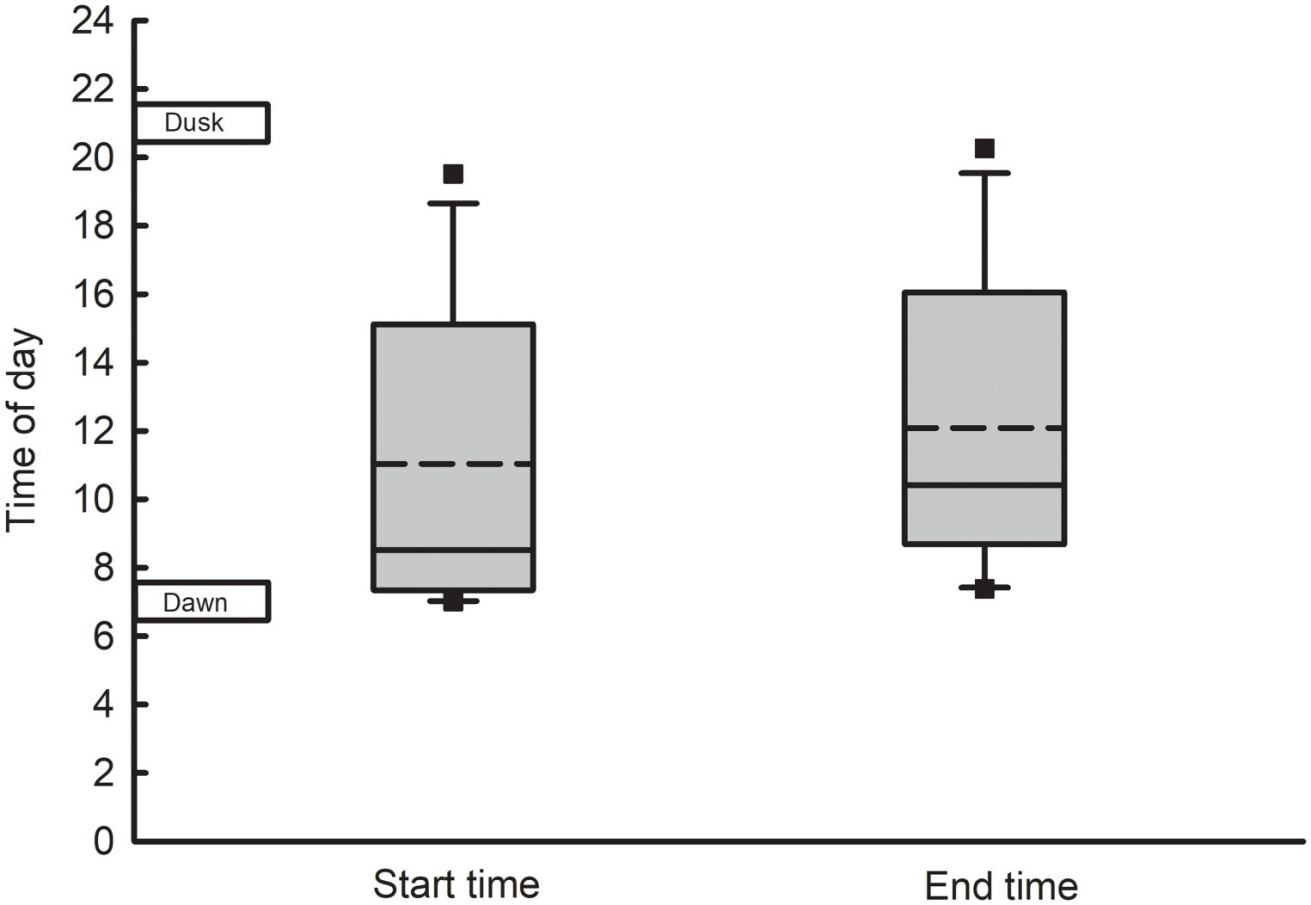
Time of day of longest single flight of western corn rootworm females on flight mills. Includes only females whose longest flight was ≥10 min. Dawn is marked at 7 h and dusk is marked at 21 h. The box boundary closest to the x-axis indicates the 25th percentile, solid line within box marks the median, dashed line within box marks the mean, and the boundary farthest from the x-axis indicates the 75th percentile. Whiskers above and below the box indicate the 95th and 5th percentiles, respectively, and outliers are represented as squares.

Distance of total flight, sum of all flight distances during the 22 h testing period, was significantly affected by larval rearing density, but distance of the longest single flight was not (Fig 4A; Table 2). Additionally, total flight duration and duration of the longest single flight were significantly affected by larval rearing density (Fig 4B; Table 2). The means for total flight distance and duration were less than those for longest single flight because of the 10 min threshold requirement for being included in the longest single flight. In other words, there was a large number of short flights lasting <10 min which depressed the means for total flight. There was no effect of larval rearing density on flight speed (Table 2). Flight periodicity of the longest single flight occurred exclusively between dawn and dusk. Female beetles began their longest flight, on average, at approximately 11:00 h, and ended it, on average, at approximately 12:00 h (Fig 5). Approximately 40% of females began their longest flight at 07:00 h, which was the start of the light period (daytime). No flight lasting >10 min occurred during the dark period (nighttime).

**Fig 6:**
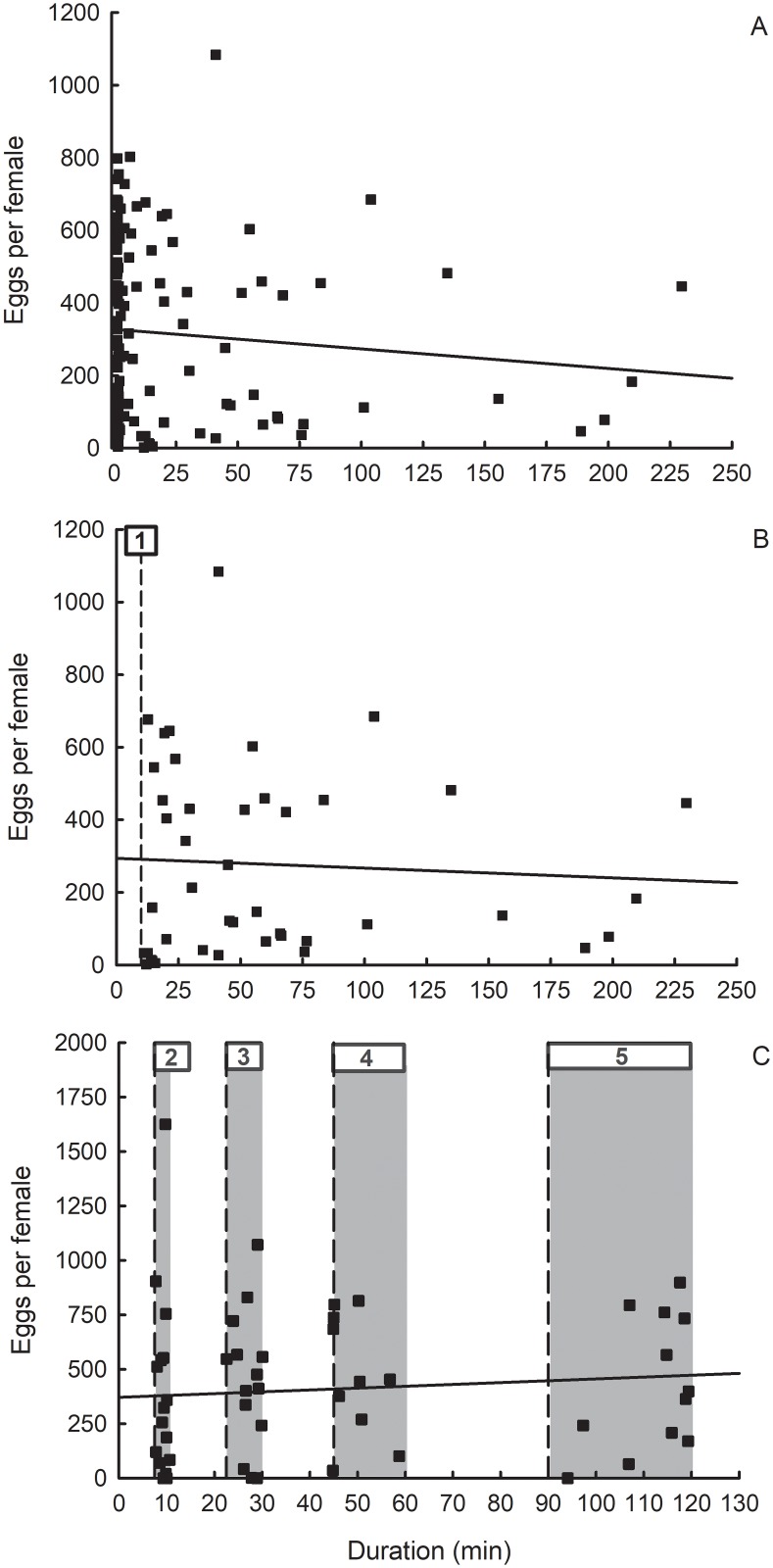
Influence of longest single flight duration on lifetime fecundity of western corn rootworm in (A, B) voluntary flight experiment, and (C) forced flight experiment. **(A)** Linear regression of eggs per female (Y) on the duration of the longest single flight (X): df = 1, 126, Y = -0.54X + 327.66, t = -1.08, P = 0.2835, r^2^ = 0.0091. (B) (1) Dashed line at 10 min indicates separation between trivial fliers and sustained fliers. Linear regression of eggs per female (Y) on the duration of the longest single flight (X) for females that engaged in a single flight lasting ≥10 min: df = 1, 43, Y = -0.22X + 244.93, t = -0.31, P = 0.7548, r^2^ = 0.0023. (C) Shaded regions span the minimum duration to intended duration for each treatment group: (2) 7.5 to 10 min, (3) 22.5 to 30 min, (4) 45 to 60 min, and (5) 90 to 120 min. Linear regression of eggs per female (Y) on the duration of the longest single flight (X): df = 1, 53, Y = 0.73X + 383.45, t = 0.54, P = 0.5907, r^2^ = 0.0055.

Regression analyses indicated that lifetime fecundity was unaffected by total time engaged in flight (Fig 6). For the voluntary flight experiments, there was no relationship between the longest single flight on flight mills and lifetime fecundity, whether all flights ≥1 min (Fig 6A; Table 3) or ≥10 min (Fig 6B; Table 3) were considered. There was no effect of forced flight, at the limited durations tested, on lifetime fecundity (Fig 6C; Table 3). Furthermore, there was no difference in longevity or fecundity between flight-tested females and controls (Table 4). Under the conditions of our study, we found no evidence, in either the voluntary or forced flight experiments, of a tradeoff between flight and egg production.

**Table 3:** Summary of linear regression analyses of flight vs. fecundity.

X	Y	df	*a ± SE	t	P	r^2^
Longest single flight duration	Eggs per female^a^	1, 126	-0.54 ± 0.50	-1.07	0.2855	0.0090
Longest single flight duration	Eggs per female^b^	1, 43	-0.22 ± 0.69	-0.31	0.7548	0.0023
Total flight duration	Eggs per female^c^	1, 53	0.73 ± 1.35	0.54	0.5907	0.0055

*a = estimated slope and SE = standard error

^a^Females from voluntary flight experiments that made a flight ≥1 min

^b^Females from voluntary flight experiments that made a flight ≥10 min

^c^Females from forced flight experiments

**Table 4:** Mean female longevity and egg number of control and tested female western corn rootworm from forced flight experiments (Mean ± SE).

	df	Control	Experimental	t	P
Longevity (days)	119	57.68 ± 3.28	58.69 ± 2.98	0.221	0.8256
Egg number	119	385.38 ± 35.40	398.22 ± 33.55	0.255	0.7961

## Discussion

Larval population density is one of the principal factors that induces facultative migratory behavior in insects [50]. Examples include *Mamestra brassicae* (Lepidoptera: Noctuidae) [51], *Spodoptera exempta* (Lepidoptera: Noctuidae) [52], *Mythimna separata* (Lepidoptera: Noctuidae) [53], and *Loxostega sticticalis* (Lepidoptera: Pyralidae) [54], in which flight activity was significantly greater for adults reared at high larval densities. Likewise, our results suggest that female western corn rootworm emerging from high density populations in cornfields are more likely to disperse and disperse farther than those emerging from low density populations.

In this study, flight duration was not associated with effects on lifetime fecundity. In general, long-distance flight is a costly behavior that may force a tradeoff with adult reproduction [40, 55]. This tradeoff can manifest as a decrease in reproductive output caused by reduced longevity and egg production. Such tradeoffs have been observed in some insects, especially hemimetabolous species. For example, extended flight decreased fecundity and fertility in *Prokelisia dolus* (Hemiptera: Delphacidae) (Wilson) [56] and *Gryllus rubens* (Orthoptera: Gryllidae) [57]. However, we found no evidence of a tradeoff in flight activity and female fecundity for western corn rootworm. This may be because flight activity was restricted to a single day. Nevertheless, some individuals flew substantial distances, up to 16.5 km during the 22 h test period, with the longest continuous flight reaching 7.7 km. Energetic costs of flight may have been offset by subsequent adult feeding as well.

Determining spatial dimensions over which tactics must be applied to mitigate local development of resistance to Bt corn or other control measures, often referred to as a resistance hotspot, in a western corn rootworm population will depend on a better understanding of dispersal [58]. Mitigation measures will not be successful if they are restricted to too small of a spatial scale around a resistance hotspot, because resistant adults will disperse beyond the mitigation area [18]. Our results indicating that rootworm larval density influences the propensity of females to engage in long-distance flight has potentially important implications for managing resistance. For example, adult dispersal from a field of Bt corn where the population is resistant or partially resistant to the Bt toxins may be greater than from a similar Bt cornfield with a more susceptible population, because larval densities will be greater in the former. This means that determining adequate dimensions of a mitigation area around a resistance hotspot must take larval density into account or at least be based on a worst case scenario of a field harboring a high-density population.

In a tethered flight study of western corn rootworm by Naranjo [35], duration of sustained flight (≥20 min) by 5-day old females was not significantly affected by larval density. Larval density significantly affected duration of trivial flights (≤20 min) by 25 day old females but not 5 day old females. There was a curvilinear response of trivial flight duration to larval population density, with flight activity being greatest at the moderate density. The differences in Naranjo’s [35] results and ours may be due to methodology. Naranjo defined sustained flights as lasting ≥20 min, whereas we placed this threshold at 10 min. Additionally, we tested only 6 day old females. In earlier tethered flight studies, sustained flight was observed among younger, mated females, and no sustained flights were taken by females older than 6 [34], or 9 days [49]. We infested seedlings with specific numbers of 7 day old larvae to establish density treatments, whereas Naranjo used egg densities to generate differential relative larval densities that could not be strictly controlled or known. Although egg densities influenced adult size, the proportion of viable eggs used in generating treatment groups was unknown. Survival in natural populations from egg to adult varies among years, ranging from 6.7% to 11% [59]. Furthermore, the percentage adult emergence from artificial egg infestations in the field can vary from 0.8% to 9% [36]. These high levels of mortality may introduce substantial stochastic variation into the experimental design. The use of 7 day old larvae to create treatment groups allowed us to impose tighter control over larval density, but the tradeoff is that we could not test the effect of density during the first 7 days of larval development.

The effects of larval crowding may influence the population dynamics of western corn rootworm through changes in development and survival. The idea that larval stress reduces adult fitness has been examined for many insects, including western corn rootworm. In greenhouse experiments, increasing larval density of western corn rootworm delayed development, reduced adult size, and increased adult mortality [60]. Increasing egg densities in field studies reduced larval development rate and adult size [36–38]. Onstad et al. [61] developed models from published data that describe density-dependent effects on survival from egg to adult. The successful imposition of a density-induced stress gradient on larvae reared in our study, as evidenced by a significant decrease in adult emergence (Fig 1) and size (Fig 2) with increasing density, implies that our three density treatments were appropriate for testing their effects on flight.

The distribution of all flights was highly skewed, with 70% lasting < 3 min (Fig 3). Frequency declined rapidly with increasing flight duration, consistent with previous tethered flight studies of western corn rootworm [34, 47, 49], and as commonly observed in many species including *Amyelois transitella* (Lepidoptera: Pyralidae) [62], *Ostrinia nubilalis* (Lepidoptera: Crambidae) [63], and *Agrilus planipennis* (Coleoptera: Buprestidae) [64]. Our data suggest that the majority of female rootworms engage in numerous intrafield flights while a small proportion engage in longer interfield flights. There was no clear break in the distribution to separate trivial and sustained flights, as seen at 20 min by Naranjo [49] and 30 min by Coats et al. [34]. Despite the lack of a clear separation, flight frequency had decreased considerably by 10 min in our study (Fig 3), as was the case for methyl-parathion susceptible (control) females in the tethered flight study by Stebbing et al. [47]. Furthermore, the long break observed by Coats et al. [34] began around 10 min. In our study, flights ≥10 min occurred exclusively during the daylight period, with peak sustained flight activity greatest during early morning, and evening hours. Interfield movement typically occurs during the morning and evening [26]. Witkowski et al. [25] inferred from beetle counts on sticky traps that flight activity in cornfields was bimodal, with flight peaks during the hours just before sunset and just after sunrise. These hours may permit greater control over flight direction, as these are times of reduced wind [65].

In conclusion, our findings have a number of implications for resistance management of western corn rootworm. Our study was not designed to determine dispersal distances of adults reared under different larval densities. Instead, the importance of our findings are in their implications for differential relative dispersal rates caused by different population densities in the landscape. Spatial differences in larval densities can result from the spatial mosaic of non-Bt corn and Bt corn, use of soil insecticides, crop rotation, distribution of Bt-resistant populations relative to fields planted to Bt corn, and other factors affecting egg or larval mortality or attractiveness of a field to ovipositing females. It will be important to explore the magnitude of any effects of differential dispersal rates on gene flow over space and on resistance management strategies. This could be done through sensitivity analyses of different larval density scenarios in models of resistance evolution. Future insect resistance management (IRM) models should test differential effects of larval densities on IRM strategies by incorporating multiple dispersal rates. Our other important finding is the apparent lack of a tradeoff between flight activity and female fecundity. Because only one day of flight activity was tested, an effect of flight activity over multiple days cannot be ruled out, but so far the evidence suggests modelers may not need to worry about the effects of different rates of dispersal on fecundity. Finally, there is evidence that male dispersal behavior differs from that of females, and the response to larval density may differ as well. A full understanding of the role of long-distance dispersal on gene flow will also require examination of male flight behavior.

## Supporting information

S1 Fig Insect flight mills used for tethered flight experiments. (A) Entire insect flight mill and (B) working portion of the flight mill. (A) Working portion of the flight mill is circled, (B) (1) 31.8 cm hypodermic tube flight arm, (2, 3) repelling ferrite ring magnets, (4) digital Hall effect sensor, (5) small nickel ring magnet used to trigger the sensor, and (6) hypodermic thin wall tube ("central pin") that separates the repelling magnets (2,3). Flight mill design modified slightly from original design of Jones et al. [46].
(TIF)
