# Supplementary figures and images for: Effects of larval density on dispersal and fecundity of western corn rootworm, Diabrotica virgifera virgifera LeConte (Coleoptera: Chrysomelidae)
Source: PLoS One. 2019 Mar 1;14(3):e0212696. doi: 10.1371/journal.pone.0212696 (PMC6396902; doi:10.1371/journal.pone.0212696)

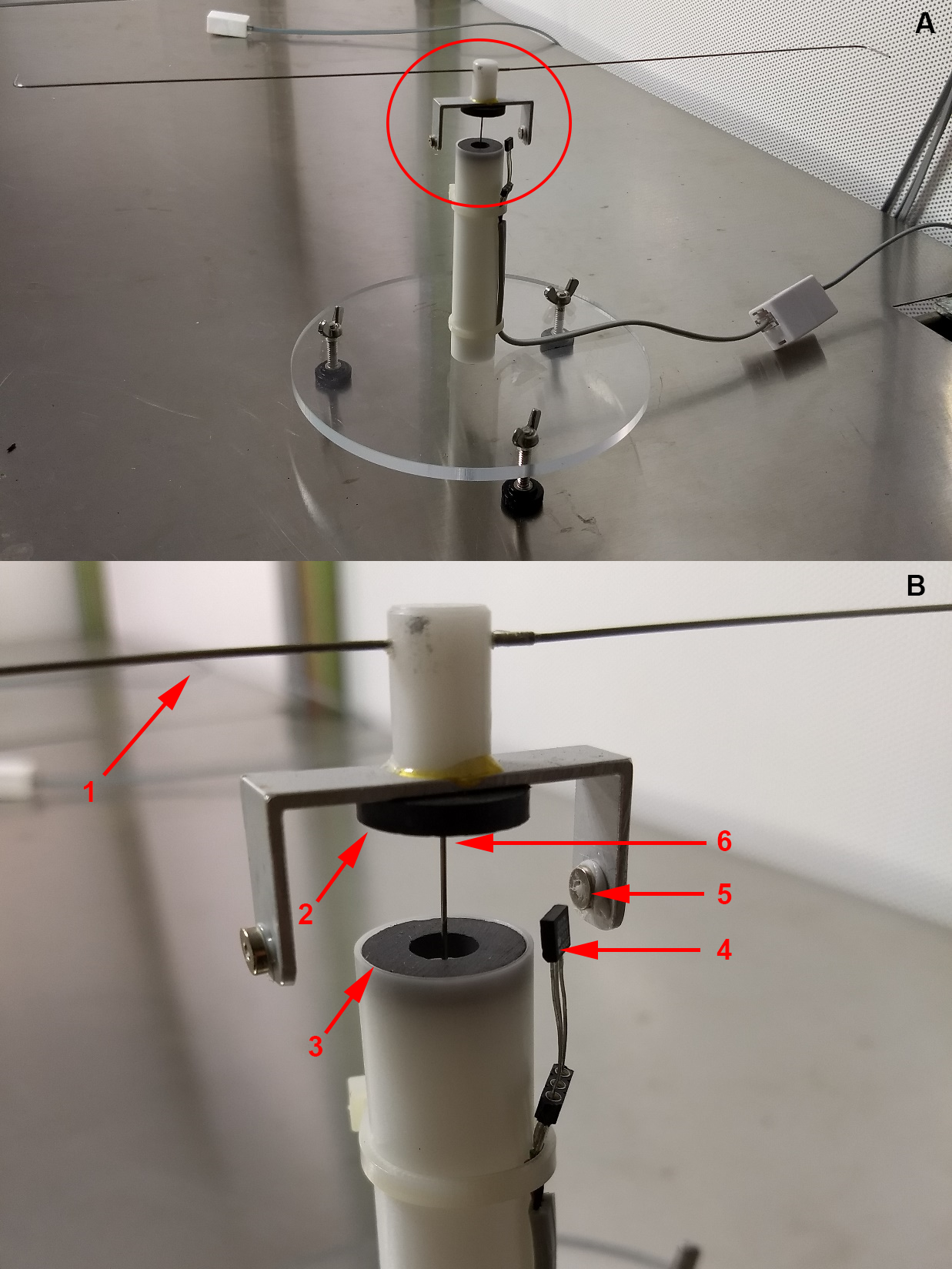

Supplement: S1 Fig — (A) Entire insect flight mill and (B) working portion of the flight mill. (A) Working portion of the flight mill is circled, (B) (1) 31.8 cm hypodermic tube flight arm, (2, 3) repelling ferrite ring magnets, (4) digital Hall effect sensor, (5) small nickel ring magnet used to trigger the sensor, and (6) hypodermic thin wall tube ("central pin") that separates the repelling magnets (2,3). Flight mill design modified slightly from original design of Jones et al. [46]. (TIF) [file pone.0212696.s001.tif]
